# Comparison between Golnar product and placebo in heavy menstrual bleeding: A double-blind randomized clinical trial

**Published:** 2020

**Authors:** Fatemeh Yousefi, Maryam Kashanian, Ismail Nazem, Soodabeh Bioos, Omid Sadeghpour, Jale Aliasl, Fataneh Hashem-Dabaghian

**Affiliations:** 1 *Research Institute for Islamic and Complementary Medicine, School of Persian Medicine, Iran University of Medical Sciences, Tehran, Iran*; 2 *Department of Obstetrics and Gynecology, Iran University of Medical Science, Tehran, Iran*; 3 *Department of Persian Medicine, School of Persian Medicine, Tehran University of Medical Science, Tehran, Iran*; 4 *Traditional Medicine Clinical Trial Research Center, Shahed University of Medical Sciences, Tehran, Iran*

**Keywords:** Persian medicine, Herbal medicine, Uterine hemorrhage, Menorrhagia, Clinical trial

## Abstract

**Objective:**

Golnar product is a poly herbal formulation advised by Persian medicine to control heavy menstrual bleeding (HMB). This study was conducted to compare the efficacy of this product with placebo in patients with HMB.

**Materials and Methods:**

In this double-blind randomized clinical trial, 100 women with HMB were randomly assigned into two groups. The patients in the Golnar group (n=50) took Golnar capsules 500 mg three times a day for the first 7 days of menstrual cycle for three cycles. The placebo group (n=50), took placebo capsules in the same manner.

The duration and volume of bleeding (using Pictorial Blood Loss Assessment Chart: PBAC), quality of life (using Menorrhagia Questionnaire: MQ), and hemoglobin level (Hb) were measured 3 months after initiation of the intervention.

**Results:**

Eighty-two patients (43 in the Golnar and 39 in the placebo groups) completed the 3-month intervention period. In the Golnar group, PBAC score decreased from 201.62 (144.11) to 109.44 (69.57) (p<0.001) and MQ score improved significantly from 0.58 (0.27) to 0.39 (0.31) (p<0.001), while changes in placebo group were not significant. Hb increased in the Golnar group from 12.78±0.98 to 12.97±0.95 mg/dl (p=0.048) and decreased in the placebo group from 12.94±1.08 to 12.44±1.01mg/dl (p<0.001). No significant adverse effects were found in the Golnar group.

**Conclusion:**

The Golnar product can be considered an effective intervention for patients with HMB. Assessment of side-effects is suggested to be performed in a larger sample. In addition, a comparison between the Golnar product and nonsteroidal anti-inflammatory drugs could be valuable.

## Introduction

Heavy menstrual bleeding (HMB) is a major problem (Kiran et al., 2018 ▶; Bruinvels et al., 2016 ▶) with the prevalence of 20% in women of childbearing age (Kiran et al., 2018 ▶; Azizkhani et al., 2018 ▶). HMB can be diagnosed based on excessive (more than 80 ml) or prolonged (more than 7 days) uterine bleeding per menstrual cycle (Tower and Frishman, 2013 ▶; Hoffman et al., 2012 ▶). The negative effects of HMB on the quality of life and sexual activity as well as the development of iron deficiency anemia compel the patients to look for treatment which may impose substantial expenses (De Souza et al., 2010 ▶; Matteson et al., 2013 ▶). 

Conventionally, some pharmacological and non-pharmacological treatments have been recommended for this disorder, some of which have side-effects or are contraindicated in some patients (Gibbs et al., 2008 ▶; Chen et al., 2015 ▶; Bradley and Gueye, 2016). 

Nowadays, complementary and alternative therapies have become very popular (Zhang et al., 2015 ▶). 

Masters of Persian medicine believed that normal menstruation is an important sign of a woman's health and recommended valuable guidelines for the diagnosis and treatment of menstrual disorders (Ibn Sina, 2005 ▶; Jorjani, 2005 ▶). 

Based on the Persian medicine literature, HMB is equal to “*Effrate-Tams*” or “*Kasrat-e-Tams*” and is described in three categories (Jorjani, 2005 ▶; Arzani, 2010 ▶; Aghili-Khorasani, 2008 ▶):

1. High volume of menstrual bleeding in every period.

2. Continued bleeding after completion of the menstrual period.

 3. Bleeding in the intervals between menstrual periods.

In Persian medicine, several medicinal plants were introduced to treat hypermenorrhea (Mobli et al., 2015 ▶). 

As a herbal complex, Golnar product consists of six medicinal ingredients including the flowers of *Punica granatum *L or Persian Golnar (Lythraceae), which is the main ingredient, the gum of *Acacia arabica (*Leguminosae)*, Armenian bole *(a mixture of iron oxide), the flowers of* Rosa damascena (*Rosaceae*)*, *Astragalus gossypinus *Fisch.* or *tragacanth (Leguminosae), and bark of *Cinnamomum aromaticum *J Graham (Lauraceae) (Abuali, 2009 ▶; Hosseini-Shafaei, 2004 ▶).

Based on Persian medicine, *Punica granatum* is cold and dry in nature and considered an astringent (Abdul et al., 2013 ▶). Scientific studies demonstrated that this plant has an inhibitory effect on prostaglandin synthesis (Fathima and Sultan, 2012 ▶; Mobli et al., 2015 ▶). 

In the quasi-experimental study done by Memarzadeh et al. (2015) ▶ to assess the efficacy of *P. granatum* in HMB, bleeding volume was significantly reduced. 

Goshtasbi et al. (2015) ▶ also compared the effect of *P. granatum* with tranexamic acid in HMB and concluded that both interventions reduced the bleeding volume. 

Imdad et al. (2017) ▶ compared the efficacy of Golnar tablet (including *Punica granatum, Acacia arabica, Armenian bole *and* Rosa damascena*) with tranexamic acid in HMB. The results showed the efficacy of both interventions. 

*Rosa damascena* is also posited as an astringent plant with pharmacological properties for menstrual bleeding as demonstrated in a comprehensive review (Boskabady et al., 2011 ▶).

*Cinnamomum aromaticum *is rich in tannin (Morimoto et al., 1986 ▶) and has astringent properties (Lee and Ahn, 1998 ▶).

Bolus Armenia Rubra or *Armenian bole *is used in Unani medicine in combination with other drugs to treat uterine bleeding (Imdad et al., 2017 ▶). The polymeric components in the aqueous extracts of gum acacia (*Acacia arabica)* shorten the activated partial thromboplastin time (aPTT) and prothrombin time (PT). These components have hemostatic effects and accelerate blood coagulation (Bhantnagar et al., 2013 ▶). It is used to treat hemoptysis, and menorrhagia (Jahan, 2008 ▶). 

Tragacanth is used as a modifier for some side effects of many herbal drugs in Persian medicine. Modifiers are plants that reduce or remove the unpleasant effects of other drugs (Aghili Khorasani, 2011 ▶).

There has been no trial on the efficacy and safety of the Golnar product although Persian medicine specialists administer it. Thus, this clinical trial compared the effects of the Golnar product and placebo on the volume and duration of uterine bleeding in patients with HMB. 

## Materials and Methods

**Study design**

This randomized, double-blind, placebo-controlled trial was performed in Qom province (Iran) from January 2017 to February 2018. The protocol was approved by the ethics committee of Iran University of Medical Sciences (IR.IUMS.REC.139509221309201) and registered in the Iranian registry of clinical trials with the identifier of IRCT2017010231722N1. 

In this study, 100 patients were selected according to the following inclusion criteria: 

Age from 20 to 45 years, a menstrual period longer than 7 days, menstrual bleeding volume more than 80 ml, PBAC (Pictorial Blood Loss Assessment Chart) score over 100, endometrial line 12 mm or less, normal Pap smear, hemoglobin 11 mg/dl or greater, normal gynecological observation, not being pregnant, not lactating, and not receiving hormone therapy. 

The patients were excluded if they had a history of significant medical problems (coagulopathies, diabetes mellitus, chronic inflammatory disease, and thyroid dysfunctions); endometrial abnormalities such as hyperplasia, cervical carcinoma, uterine or ovary malignancy; sub-mucosal or intramural fibroids larger than 5 cm; systemic or pelvic inflammatory diseases; known hyperprolactinemia; or hemoglobin above 16 mg/dl. Other exclusion criteria involved intention to become pregnant in 3 months, surgery, and emergency procedure because of increased bleeding during the study, the use of any herbal or chemical agent interfering with the drug studied or affecting menstrual bleeding such as oral contraceptive pills, and non-adherence to use this medication. All of the subjects were free to withdraw at any time during the course of the study.

At the baseline, the patients were evaluated for demographic characteristics, complete history, physical examination, uterine sonography, pap smear, complete blood count (CBC), thyroid stimulating hormone (TSH), partial thromboplastin time (PTT), prothrombin time (PT), and serum prolactin. The volume of bleeding was measured using the Pictorial Blood Loss Assessment Chart (PBAC) and the quality of life was assessed using the menorrhagia questionnaire (MQ). At the end of the study, the patients completed the PBAC, MQ, and a checklist of side-effects. CBC was repeated at the end of the study to evaluate hemoglobin changes. 

**Randomization**

After signing a written informed consent, the patients were randomly assigned into two groups of placebo (n=50) and Golnar product (n=50). Randomization was performed through the block randomization method in which all possible combinations of AABB were selected according to computer-generated random numbers. To conceal the randomization process, a random list was generated and used by a blinded practitioner who enrolled the participants. 

**Plant material**

The drug is produced and registered by “Tuba Green Gold” Company (registration code: 6485218733900485).

*Punica granatum* and* Rosa damascena *flowers (9.54 g of each) were used along with the gums of* Acacia arabica and Armenian bole, *and bark of *Cinnamomum aromaticum* (12.72 g of each material), and the gum of *Astragalus gumifer* (6.36 g).

The drug components were purchased from the local market and authenticated at the herbarium of *Talay*-*e-sabz-e Tuba* Company. Voucher specimen for five plants were deposited at the Herbarium of Tehran University of medical sciences: *Punica granatum* L (Lythraceae) PMP-541, *Rosa damascena Herrm *(Rosaceae) PMP-540, *Acacia Senegal wild *(Leguminosae) PMP-857, *Cinnamomum verum *(Lauraceae) PMP-917, *Astragalus gossypinus *Fisch (Leguminosae) PMP-856. The sixth component, *Armenian bole*, was a mineral. The components were powdered, mixed, and soaked in Golnar water (macerate) and subsequently dried by an oven at 60-70°C. To prepare Golnar water, each 10 g of Golnar powder was soaked using 100 ml water such that 10% of the total combination was Golnar water (Abuali et al., 2009 ▶; Hoseini Shafaei, 2004 ▶). 

Afterward, 500 mg capsules were filled with the prepared materials. Powdered rusks as a neutral material for bleeding were used to prepare the placebo (500 mg capsules). Capsules and containers were similar in shape and color. 

Golnar capsules were standardized based on total phenols (Folin- Ciocalteau method) content. Each capsule contained polyphenol 70.05 mg/g. 

**Assessment of uterine bleeding**

Uterine bleeding was evaluated by the PBAC (Pictorial Blood Loss Assessment Chart), an instrument which objectively estimates menstrual blood loss (Higham et al, 1990 ▶). In this method, the number of used pads or towels and the degree to which they are stained with blood, are recorded. Scores above 100 indicate that the bleeding volume has been greater than 80 ml. The participants were asked to record this chart before and after the end of the study. Sensitivity and specificity of the PBAC in identifying menorrhagia are 80 and 88%, respectively (versus the alkaline hematin method as the most valid technique) (Higham et al, 1990 ▶). Johnson and colleagues validated this chart and the values of positive and negative tests were 85.9 and 84.8% of the expected value, respectively (Janssen et al., 1995 ▶). 

Quality of life was evaluated by the MQ. This questionnaire was developed by Ruta *et al.* in 1996 (Ruta et al., 1995 ▶). Cronbach alpha coefficients for the Persian version of MQ were 0.73 for the first administration and 0.77 for the second administration (Mazari et al., 2012 ▶).

**Intervention**

The participants were asked to take three 500 mg capsules (Golnar product or placebo) 20 min after every meal for the first 7 days of menstruation and follow the pattern for 3 cycles. They were not allowed to use any drug except for acetaminophen, nor were permitted to use mefenamic acid, tranexamic acid, hormonal therapy, herbal medicine, or medicinal herbs during the study. 

**Safety assessment**

In order to detect possible side effects, all the patients were followed by physicians every two weeks and asked to report any side effects.

**Sample size**

The formula for comparing means was used to calculate the sample size. A total of 45 patients per group was calculated considering the type I error of 0.05, study power of 80%, attrition rate of 20%, and the information observed in the pilot study (standard deviation=144 and 90 in each group; difference of PBAC between groups=80).

**Statistical methods**

Per-protocol analysis was performed using the SPSS software (version 17). Qualitative variables were captured as frequency and compared between groups using the Chi-square test. Quantitative variables are presented as mean (±standard deviation) or median (interquartile range) and compared between groups using student t-test. Paired t-test was employed to measure the changes of variables in each group. A p-value<0.05 was considered significant.

## Results

**Figure 1:**
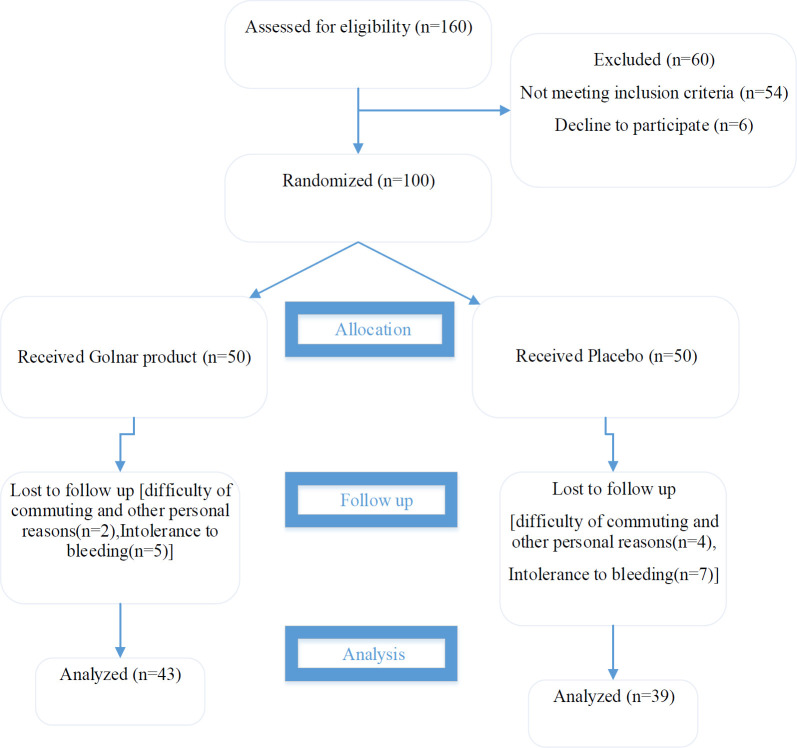
The consort flow diagram

Figure 1 displays the consort flow diagram of the study. A total of 82 patients (43 (52.4%) in the Golnar group and 39 (47.6%) in the placebo group) completed the 3-month intervention. Attrition was statistically identical between the groups (p=0.2).

**Baseline and demographic characteristics**

The baseline characteristics are presented in Table 1. The frequencies of age, education level, marital status, duration of disease, type of childbirth, the number of children and hemoglobin level were similar between the groups. 

**Figure 2.**
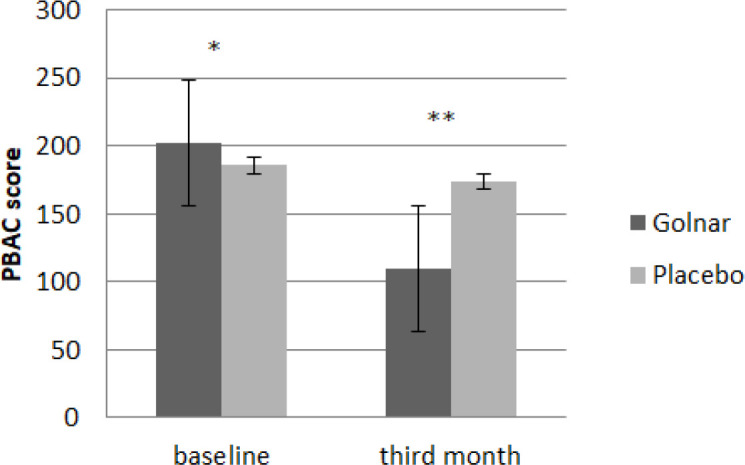
PBAC changes in the study (* p=0.54, ** p<0.001).

The volume and duration of bleeding, MQ score and hemoglobin level before and after interventions are presented in Table 2 and Figure 2 presents the PBAC changes during the study.

**Volume of bleeding**

At baseline, the volume of bleeding (PBAC score) was statistically similar between the groups (p=0.54), but it was significantly different at the end of the 3rd month of the study (p<0.001). 

 The volume of bleeding decreased as the amount of 38.04±29.1% in the Golnar group (p<0.001) in comparison with 3.71±32.2% in the placebo group (p=0.31). At the end of the study, 53 patients (20 (37.7%) in the Golnar group and 33 (62.3%) in the placebo group) had the PBAC score more than 100 (volume of bleeding more than 80 ml) (p<0.001).

**Duration of bleeding**

At baseline, duration of bleeding was statistically similar between the groups (p=0.26), but at the end of the study, it was significantly lower in the Golnar group than the placebo group (p=0.004). 

Duration of bleeding (day) was reduced about 15.9±20.3% in the Golnar group (p<0.001) while increased about 5.6±26.6% in the placebo group (p=0.54).

**Table 1:** Baseline characteristics of the participants

**variables **	**group**		**p value**
Age (year) (mean±SD)	Golnar	33.39±8.03	0.75*
	placebo	33.89±6.5	
Education n (%)	Golnar	<12 years: 31(72.1)	0.07***
		>12 years: 12 (27.9)	
	placebo	<12 years: 20 (51.3)	
		>12 years: 19 (48.7)	
Marital status n (%)	Golnar	single: 6 (14)	0.62***
		married: 37 (86)	
	placebo	single: 5 (12.8)	
		married: 34 (87.2)	
Duration of disease (month) (mean±SD)	Golnar	29.13±28.27	0.53*
	placebo	33.33±32.32	
Number of children median (IQR)	Golnar	2 (1-3)	0.72**
	placebo	2 (1-2.25)	
Type of childbirth n (%)	Golnar	normal: 16 (59.3)	0.19***
		CS: 8 (29.6)	
		both: 3 (11.1)	
	placebo	normal: 9 (34.6)	
		CS:13 (50)	
		both: 4 (15.4)	
Hemoglobin (g/dl) (mean±SD)	Golnar	12.78 (0.98)	0.49*
	placebo	12.94 (1.08)	

*T-test, **Mann-Whitney U test, ***Chi-square test, SD: standard deviation, IQR: interquartile range, CS: cesarean section

**Table 2:** PBAC and MQ scores before and after the intervention

variable	Group	before intervention Mean±SD	after intervention Mean±SD	p value*
PBAC score	Golnar	201.62±144.11	109.44±69.57	<0.001
	Placebo	185.58±87.74	173.48±87.14	0.31
	p value**	0.54	<0.001	
Duration of bleeding (day)	Golnar	9.3±2.8	7.5±1.7	<0.001
	Placebo	8.6±2.5	8.8±2.4	0.54
	p value	0.26	0.004	
MQ score	Golnar	0.58±0.27	0.39±0.31	<0.001
	Placebo	0.57±0.31	0.56±0.33	0.541
	p value	0.89	0.018	
Hemoglobin (mg/dl)	Golnar	12.78±0.98	12.97±0.95	0.048
	Placebo	12.94±1.08	12.44±1.01	<0.001
	p value	0.49	0.018	

Paired t-test, ** t-test, SD: standard deviation

**MQ score**

At baseline, the MQ score was statistically similar between the groups (p=0.89), but it was significantly different at the end of the study (p=0.01). 

Quality of life was improved in the Golnar group (p<0.001), but was not significantly changed in the placebo group (p=0.54).

**Hemoglobin**

At baseline, Hb was statistically similar between the groups (p=0.49), but it was significantly different at the end of the study (p=0.01). Hb was significantly increased in the Golnar group (p=0.04), but decreased in the control group (p<0.001).

**Side effects**

Of the 87 patients who used the medications for at least one cycle, 21 (24%) patients experienced some adverse events in both groups. In the Golnar group, 13 (26%) women reported symptoms including abdominal pain, gastrointestinal reflux, constipation, dryness of skin, excessive appetite, and leukorrhea. In the placebo group, 8 (16%) patients experienced abdominal pain, constipation, bloating, heartburn, and dryness of mouth. The difference between groups was not statistically significant (p=0.3). 

## Discussion

The results of this study indicated the efficacy of the Golnar product in HMB without significant side effects. The volume of bleeding was reduced and the quality of life improved significantly in the intervention group after 3 months. In addition, the hemoglobin level was significantly elevated in the Golnar group, while it diminished in the placebo group. Women in the Golnar group reported more side effects; however, the difference between the groups was not statistically significant. Further, there was no attrition due to side effects, and all unpleasant experiences were tolerated. 

The results of the present study are similar to those reported in the study by Imdad et al. (2017) ▶, where PBAC score and quality of life were compared between Golnar tablet and tranexamic acid. Four components of Golnar tablet including *Punica granatum, Acacia arabica, Armenian bole*, and* Rosa damascena *are similar to those of the Golnar product in our study. In the mentioned study, the duration of treatment was 4 days from the onset of bleeding, with the pattern followed for 3 cycles. PBAC and quality of life scores significantly diminished in both groups. Nonetheless, hemoglobin changes were not measured, and the study was single blind. 

Memarzadeh et al. (2015) ▶ assessed the volume of bleeding and the quality of life in patients with uterine leiomyoma. In this study, the participants received 5 ml of the *Punica granatum* syrup three times a day for 7 days from the onset of HMB and were followed for three cycles. Bleeding was significantly reduced compared to the baseline. Lack of a control group was one of the limitations of this study. 

Goshtasbi et al. (2015) ▶ compared the effect of *Punica granatum* and tranexamic acid on HMB. A 500-mg dose of the dried powder of *Punica granatum *flowers was administered every 6 hr for five consecutive days from the first day of menses and followed for 3 cycles. In the same manner, tranexamic acid 500 mg was given on a daily basis. Although the volume of bleeding dropped in both groups, there was no statistically significant difference between the groups. In addition, quality of life improvement was not significantly different between the two groups. 

The vasodilatory effects of prostaglandin E2 and I2 as well as the antiplatelet aggregation activity of prostaglandin I2 play a significant role in extreme bleeding during menstruation (Livingstone and Frase, 2002 ▶).

The plants that control abnormal uterine bleeding work through four mechanisms: inhibiting inflammatory processes, inhibiting prostaglandins production, antiproliferative activity on human cervical cancer cells, and estrogenic activity (Mobli et al., 2015 ▶). 

Golnar inhibits biosynthesis of prostaglandins and its flowers have anti-inflammatory and antiproliferative activity (Mobli et al., 2015 ▶; Livingstone and Frase, 2002 ▶). 

Because of the existence of tannins such as gallic acid, ursolic acid, triterpenoids including maslinic and asiatic acid in Golnar flowers, it has astringent activity and is known as a hemostatic drug. It is employed in different forms such as extract, powder, syrup, nasal drop, brewed drink, and gargle. Previous research showed that Golnar has a significant impact on internal hemorrhages such as epistaxis, hematemesis, GI bleeding, menorrhagia, and diarrhea (Abdul et al., 2013 ▶; Nadcarni, 1954 ▶).

Other component of Golnar product is *Cinnamomum aromaticum *which has tannin and has an astringent effect (Boskabady et al., 2011 ▶; Lee and Ahn, 1998 ▶; Imdad et al., 2017 ▶; Aghili-Khorasani, 2011 ▶). *Rosa damascena, *similar to the other ingredients, is astringent; therefore, the Golnar product contains several astringent components, which reduce uterine bleeding by exerting an impact on the biosynthesis of prostaglandins.

According to Persian medicine, when there is more than one cause for a disease, it is necessary to use a complex drug to resolve the various causes. In addition, using a complex drug instead of a simple one has the following advantages of reducing the amount of medication needed, the side effects and the course of treatment along with increasing the effects of the main drug (e.g. Golnar) (Aghili-Khorasani, 2008 ▶). For example, *Tragacanth *is used to reduce the adverse effects of cinnamon (Aghili Khorasani, 2011 ▶).

Another thing to be considered is the evidence that shows the platelet anti-aggregation activity of *Cinnamomum cassia.* Although *Cinnamomum aromaticum *was used in the Golnar product and it is known to be rich in tannin (Morimoto et al., 1986 ▶), and to have astringent properties (Lee and Ahn, 1998 ▶; Aghili Khorasani, 2011 ▶), the anticoagulant effect of the Golnar product should be considered and evaluated. 

As a suggestion, further studies with larger sample sizes, longer follow-up phases with and without intervention, and comparison between the Golnar product and NSAIDs or other conventional therapies will be valuable. 

In patients with HMB, the Golnar product can reduce uterine bleeding in addition to enhancing the quality of life without significant side effects.
